# Promoter methylation of Raf kinase inhibitory protein: A significant prognostic indicator for patients with gastric adenocarcinoma

**DOI:** 10.3892/etm.2014.1833

**Published:** 2014-07-08

**Authors:** DONG-XIA LI, HAI-YANG CAI, XIA WANG, YAN-LING FENG, SONG-WANG CAI

**Affiliations:** 1 School of Basic Medical Sciences, Xinxiang Medical University, Xinxiang, Henan 453003, P.R. China; 2 The First Affiliated Hospital of Xinxiang Medical University, Weihui, Henan 453100, P.R. China; 3 Department of Pathology, Public Health Clinical Center of Fudan University, Shanghai 201508, P.R. China; 4 Department of Cardiothoracic Surgery, Third Affiliated Hospital, Sun Yat-sen University, Guangzhou, Guangdong 510630, P.R. China

**Keywords:** Raf kinase inhibitory protein, methylation, gastric adenocarcinoma, prognosis

## Abstract

DNA methylation has an important role in the development of carcinomas. As a metastasis suppressor gene, Raf kinase inhibitory protein (RKIP) suppresses tumor cell invasion and metastasis. In the present study, the associations between RKIP protein expression and promoter methylation with clinicopathological parameters, prognosis and survival rates in gastric adenocarcinoma were investigated. RKIP protein expression and promoter methylation were measured in 135 cases of surgically resected gastric adenocarcinoma specimens and corresponding normal tissues using immunohistochemistry and methylation-specific polymerase chain reaction, respectively. Kaplan-Meier analyses were performed to analyze the patient survival rate. Prognostic factors were determined using multivariate Cox analysis. RKIP promoter methylation was detected in 48.9% of gastric carcinoma tissues and 5.17% of adjacent tissues (P<0.05). RKIP protein expression was detected in 43.0% of gastric carcinoma tissues and 91.1% of adjacent tissues (P<0.05). The protein expression levels and promoter methylation of RKIP were shown to correlate with pathological staging, Union for International Cancer Control-stage, tumor differentiation and lymph node metastasis (P<0.05). In addition, the protein expression of RKIP in gastric carcinomas was demonstrated to be associated with promoter methylation of RKIP. Survival analysis of gastric carcinoma patients revealed that promoter methylation in RKIP-positive tumors correlated with a significantly shorter survival time when compared with RKIP-negative tumors (P=0.0002, using the log-rank test). Using multivariate Cox analysis, promoter methylation of RKIP was shown to be an independent prognostic factor (P=0.033). These results indicated that abnormal promoter methylation of RKIP may be one cause of downregulated RKIP expression. Downregulation of RKIP expression was shown to correlate with the incidence and development of gastric carcinomas. Thus, abnormal promoter methylation of RKIP may be a valuable biomarker for estimating gastric carcinoma prognosis.

## Introduction

The development of gastric carcinoma is associated with the abnormal expression of multiple genes (1–3), and mechanisms of abnormal gene expression include genetic alterations and epigenetic changes (4). Epigenetic changes are crucial to initiating carcinogenesis (5). DNA methylation is an important type of epigenetic change (5). DNA methylation occurs primarily in CpG islands within gene promoters, resulting in transcriptional inactivation and gene silencing (6). DNA methylation of multiple genes is associated with the development of gastric carcinoma (7–9).

Raf kinase inhibitory protein (RKIP) encodes a protein that inhibits Raf-1 (a serine/threonine kinase)-mediated phosphorylation, and thereby the activation of MEK-1 [a mitogen-activated protein (MAP) kinase kinase that specifically phosphorylates the regulatory threonine and tyrosine residues present in MAP kinases] by Raf-1 (10). The antimetastatic function of RKIP was first identified by Fu *et al* (11), and since then RKIP expression has been investigated in numerous cancer types. Downregulation of RKIP expression has been observed in a number of types of human cancer, including breast carcinoma (12), acute myeloid leukemia (13), colorectal carcinoma (14) and melanomas (15). These observations revealed that the expression of RKIP is lower in metastatic tissue compared with nonmetastatic tissue and correlated with decreased survival times, indicating that RKIP may be a prognostic marker of human cancer (16–18). The mechanisms underlying abnormal RKIP expression in gastric carcinoma remain largely unknown. Guo *et al* (19) demonstrated that RKIP methylation has an important role in the downregulation of RKIP expression in gastric cardia adenocarcinoma; however, the study did not include other subtypes of gastric adenocarcinoma. To further investigate the mechanism of RKIP expression in gastric cancer, the present study included all surgical subtypes of gastric adenocarcinoma.

In the present study, the rate of RKIP expression was determined in gastric adenocarcinoma tissues and adjacent mucosa tissues. Furthermore, the correlation between RKIP promoter methylation and RKIP expression was analyzed. The aim of the present study was to determine whether RKIP methylation may represent a valuable biomarker for estimating gastric carcinoma prognosis, and thus, may be a novel target for gastric adenocarcinoma therapy.

## Materials and methods

### Patients and tissue samples

A total of 135 patients who underwent surgical resections for primary sporadic gastric carcinoma at the First Affiliated Hospital of Xinxiang Medical University (Weihui, China) between January 2004 and December 2007 were recruited for the study. None of the patients had received preoperative chemotherapy or radiotherapy. In total, 92 males and 43 females (mean age, 58.32 years; range, 28–73 years) were included. The samples obtained included 23 cases of early gastric cancer and 112 cases of advanced gastric cancer, which constituted 31 cases of well-differentiated cancer, 48 cases of moderately differentiated cancer and 56 cases of poorly differentiated cancer. A total of 84 samples were obtained from patients with positive lymph node metastases, while 51 samples were obtained from patients with negative lymph node metastases. All the tumors were adenocarcinomas and were graded according to the World Health Organization criteria (20). Patients were followed-up until mortality or the end of the study (November 30, 2012); the mean postoperative follow-up duration was 50.76±29.70 months. The clinical and pathological characteristics of the samples obtained are shown in Table I. The study was approved by the Ethics Committee of Xinxiang Medical University and written informed consent was provided by the patient’s family,

### DNA extraction and sodium bisulfite modification

Genomic DNA was isolated using a DNA extraction kit (Tiangen Biotech Co., Ltd. (Beijing, China). The total DNA content and purity (A_260_/A_280_ >1.8) was measured using an ultraviolet spectrophotometer, and DNA integrity was analyzed using agarose electrophoresis with a gel image analysis system (Syngene, Cambridge, UK). Bisulfite conversion was performed using 1 μg genomic DNA and the CpGenome DNA Modification kit (Intergen, Purchase, NY, USA).

### Methylation-specific polymerase chain reaction (PCR)

Methylation-specific PCR (MSP) was used to analyze the methylation status of RKIP. RKIP primers were designed in accordance with the study by Al-Mulla *et al* (21) (Table II). MSP was performed in 25-μl reaction volumes. Following predegeneration at 95°C for 10 min, a total of 40 cycles of 45 sec at 95°C, 30 sec at 53°C and 30 sec at 72°C were completed, followed by a 10 min final extension at 72°C. The PCR products were subsequently observed using 2.0% agarose gel electrophoresis. A water blank was used as a negative control and placental DNA methylated by CpG methyltransferase (M.SssI), in accordance with the manufacturer’s instructions (New England Biolabs, Inc., Beverly, MA, USA), was used as a positive control.

### Immunohistochemical staining

Full tissue sections of 135 paraffin-embedded gastric adenocarcinomas were processed for immunohistochemical staining of RKIP. Specimens were fixed in 4% paraformaldehyde and embedded in paraffin. A 4-μm section from each patient was cut, dried, dewaxed and rehydrated, prior to treatment with 3% hydrogen peroxide solution for 10 min and microwave treatment in citrate buffer (pH 6.0) at 95°C (3×10 min). Nonspecific binding was blocked by treating the slides with 10% normal goat serum for 15 min. The slides were then incubated with rabbit polyclonal antibodies against human RKIP (1:200; Beijing Sequoia Jinqiao Company, Beijing, China) for 20 min at room temperature. Next, the slides were incubated with horseradish peroxidase-labeled streptavidin solution for 20 min at room temperature. Color development was achieved using a 3,3′-diaminobenzidine solution. The slides were counterstained with 1% Meyer’s hematoxylin. As a negative control for RKIP staining, tissue sections were treated with phosphate-buffered saline instead of rabbit polyclonal antibodies against human RKIP. The positive control was a normal gastric mucosa tissue known to express RKIP. Water was used instead of the primary antibody as a negative control.

### Statistical analysis

All statistical analyses were performed using SPSS software version 17.0 (SPSS, Inc., Chicago, IL, USA). The χ^2^ test and two-tailed Fisher’s exact test were used to determine the correlation between RKIP promoter methylation and expression with clinicopathological parameters. The correlation between RKIP promoter methylation and expression was assessed using the Spearman’s rank test. Kaplan-Meier analysis was used to estimate survival as a function of time, and survival differences were analyzed using the log-rank test. Multivariate analysis of the prognostic factors was performed using the Cox proportional hazards model. P<0.05 was considered to indicate a statistically significant difference.

## Results

### RKIP promoter methylation and protein expression in normal gastric tissue and gastric adenocarcinomas

**Figure 1:**
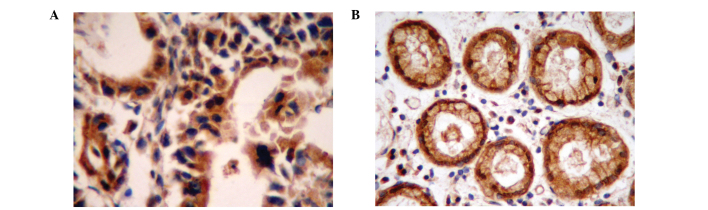
Positive immunohistochemical staining of RKIP in (A) gastric adenocarcinoma tissues and (B) normal tissues (magnification, ×400). RKIP, Raf kinase inhibitory protein.

Using the MSP method and immunohistochemical staining, RKIP promoter methylation and expression were detected in 135 pairs of gastric adenocarcinoma tissues and their matched adjacent tissues. The incidence of RKIP promoter methylation in the gastric adenocarcinoma tissues (48.9%) was significantly higher compared with the adjacent mucosa tissues (5.2%; P<0.05). RKIP protein expression was largely localized in the cytoplasm of the normal glands. In a few cells, low levels of RKIP expression were detected in the cell membranes (Fig. 1). The incidence of RKIP protein expression in the tumor tissues (43.0%, 58/135) was significantly lower compared with the adjacent mucosa tissues (91.1%, 123/135; P<0.05; Table III). A total of nine gastric adenocarcinoma cases exhibited positive RKIP expression, while the remaining 57 cases of gastric adenocarcinoma with negative RKIP expression exhibited RKIP promoter methylation. These observations indicated that low RKIP expression was associated with RKIP promoter methylation (P<0.01; Table IV).

### Correlation with clinicopathological parameters

Associations between clinicopathological parameters with the methylation status and protein expression of RKIP are presented in Table V. RKIP promoter methylation was shown to significantly correlate with the pathological staging, tumor differentiation, Union for International Cancer Control (UICC) stage and lymph node metastasis (P<0.05). No statistically significant correlations were observed between RKIP promoter methylation and age or gender (P>0.05). RKIP expression was detected in 58.1, 52.1 and 26.8% of cases in the well-differentiated, moderately differentiated and poorly differentiated groups, respectively (P<0.05). RKIP expression was demonstrated to be associated with pathological staging, UICC stage and lymph node metastasis (P<0.05), but no correlations were observed with regard to age or gender (P>0.05).

### Correlation with patient survival rates

**Figure 2:**
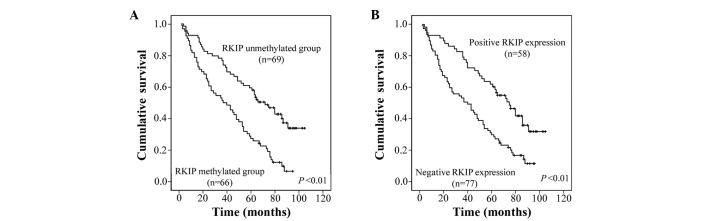
Comparison of the overall survival rates of gastric carcinoma patients with (A) RKIP promoter unmethylation vs. RKIP promoter methylation and (B) positive expression of RKIP tumors vs. negative expression of RKIP tumors. RKIP, Raf kinase inhibitory protein.

The five-year overall survival rates of the patients was 43.7%, and the median survival time was 53±5 months. The five-year overall survival rates of patients with RKIP promoter methylation were significantly lower compared with the patients with unmethylated RKIP promoters (P<0.001). Methylation of RKIP was shown to be inversely correlated with the survival of patients with gastric adenocarcinoma (Fig. 2A). The five-year overall survival rates of patients with RKIP-negative tumors were significantly lower compared with those in the RKIP-positive group (P=0.001). RKIP expression positively correlated with the survival rate of gastric adenocarcinoma patients (Fig. 2B). Furthermore, in the multivariate analysis, potential prognosis factors, including lymph node metastasis, UICC-stage and RKIP promoter methylation, were included in the Cox proportional hazards model. The results indicated that in addition to UICC-stage staging and lymph node metastasis, the RKIP promoter methylation status is an independent prognostic factor (P=0.033; Table VI).

## Discussion

RKIP plays an important role in cancer via the regulation of apoptosis induced by chemotherapeutic agents or immune-mediated stimuli, as well as functioning as a metastasis suppressor protein (22). The gene encoding RKIP is located on human chromosome 12 q24.23, and is transcribed into a 1,507-bp mRNA product containing four exons; the final protein product is 187 amino acids long. Downregulated RKIP expression activates the Raf-1/MEK/extracellular signal-regulated kinase and nuclear factor-κB signaling pathways, which enhances cell proliferation and invasion, inhibiting apoptosis and eventually leading to cancer (23).

Chatterjee *et al* (22) demonstrated that cytoplasmic RKIP was expressed at low levels in gastric cancer and directly correlated with the rate of patient survival. Similarly, in the present study, RKIP expression was demonstrated to be significantly decreased in gastric adenocarcinoma tissue, and an association with poor patient prognosis was observed. However, little information is available with regard to the mechanism responsible for RKIP downregulation in gastric cancer.

DNA methylation is one of the best-characterized epigenetic modifications and has been implicated in numerous biological processes, including transposable element silencing, genomic imprinting and X chromosome inactivation (24). Guo *et al* (25) demonstrated that RKIP promoter methylation is a major reason for the downregulation of RKIP expression. In addition, RKIP methylation was reported to lead to the downregulation of RKIP expression in gastric cardia adenocarcinoma (19). Al-Mulla *et al* (21) demonstrated that RKIP promoter methylation is a major mechanism by which RKIP expression is silenced in colorectal cancer. However, there are other mechanisms responsible for the downregulation of RKIP expression in hepatocellular carcinoma and prostate cancer. Poma *et al* (26) demonstrated that DNA methylation was unable to explain the low levels of RKIP expression observed in hepatocellular carcinoma. Furthermore, Martinho *et al* (27) demonstrated that low RKIP expression was not the result of promoter methylation in prostate cancer. However, in the present study, RKIP promoter methylation in gastric carcinoma was shown to be associated with the expression of RKIP protein; thus, was an independent prognostic factor.

In conclusion, the results of the present study demonstrated that abnormal methylation of RKIP is a crucial mechanism underlying the downregulation of RKIP expression in gastric adenocarcinoma. In addition, abnormal RKIP methylation was demonstrated to be directly associated with the progression and poor prognosis of gastric adenocarcinoma. However, the mechanisms underlying RKIP suppression of tumor cell migration and the promotion of gastric carcinogenesis by abnormal RKIP methylation remain unknown; thus, further investigation is required.

## Figures and Tables

**Table I:** Clinical and pathological characteristics of gastric adenocarcinoma cases.

Variable	Cases, n (%)
Age, years	
≥60	72 (53.3)
<60	63 (46.7)
Gender	
Male	92 (68.1)
Female	43 (31.9)
Tumor size, mm	
≥50	57 (42.2)
<50	78 (57.8)
Tumor differentiation	
Well	31 (23.0)
Moderate	48 (35.6)
Poor	56 (41.5)
Pathological staging	
Early	23 (17.0)
Advanced	112 (83.0)
Stage	
I	33 (24.4)
II	44 (32.6)
III	49 (36.3)
IV	9 (6.7)
Lymph node metastasis	
Negative	51 (37.8)
Positive	84 (62.2)

**Table II:** RKIP gene methylation specific PCR primers sequences and temperatures.

RKIP	Sequences	Length, bp	Tm, °C
Unmethylated			
Sense primer	5′-TTTAGTGATATTTTTTGAGATATGA-3′	205	53
Antisense primer	5′-CACTCCCTAACCTCTAATTAACCAA-3′		
Methylated			
Sense primer	5′-TTTAGCGATATTTTTTGAGATACGA-3′	204	53
Antisense primer	5′-GCTCCCTAACCTCTAATTAACCG-3′		

RKIP, Raf kinase inhibitory protein; Tm, melting temperature; PCR, polymerase chain reaction.

**Table III:** Methylation status and protein expression of RKIP in gastric adenocarcinoma tissues and corresponding normal tissues.

		Methylation frequency, n			Protein expression, n		
					
Tissues	Cases, n	Positive	Negative	P-value	Positive	Negative	P-value
Gastric tumor	135	66	69	<0.001	58	77	<0.001
Normal	135	7	128	<0.001	123	12	<0.001

RKIP, Raf kinase inhibitory protein.

**Table IV:** Association between RKIP methylation status and RKIP protein expression.

	Protein expression of RKIP, n		
		
Methylation status of RKIP	Positive	Negative	P-value
Methylated	9	57	<0.001
Unmethylated	49	20	-

RKIP, Raf kinase inhibitory protein.

**Table V:** Correlation between clinicopathological parameters with the methylation status and protein expression of RKIP.

Variable	Cases, n	Positive RKIP methylation, n (%)	P-value	Positive RKIP protein expression, n (%)	P-value
Age, years					
≥60	72	33 (45.8)	0.448	34 (47.2)	0.285
<60	63	33 (52.4)		24 (38.1)	
Gender					
Male	92	43 (46.7)	0.465	42 (45.7)	0.356
Female	43	23 (53.5)		16 (37.2)	
Tumor size, mm					
≥50	57	33 (57.9)	0.074	19 (33.3)	0.053
<50	78	33 (42.3)		39 (50.0)	
Tumor differentiation					
Well	31	9 (29.0)	0.037	18 (58.1)	0.005
Moderate	48	25 (52.1)		25 (52.1)	
Poor	56	32 (57.1)		15 (26.8)	
Pathological staging					
Early	23	6 (26.1)	0.016	15 (65.2)	0.018
Advanced	112	60 (53.6)		43 (38.4)	
Stage					
I	33	10 (30.3)	0.015	22 (66.7)	0.012
II	44	19 (43.2)		17 (38.6)	
III	49	31 (63.3)		17 (34.7)	
IV	9	6 (66.7)		2 (22.2)	
Lymph node metastasis					
Negative	51	19 (37.3)	0.035	28 (54.9)	0.029
Positive	84	47 (56.0)		30 (35.7)	

RKIP, Raf kinase inhibitory protein.

**Table VI:** Univariate and multivariate analyses of the disease-free survival rate.

		Univariate analysis		Multivariate analysis		
			
Variable	Cases, n	Five-year overall survival rate (%)	P-value	Relative risk	95% CI	P-value
Age, years						
≥60	72	45.8	0.352	-	-	-
<60	63	41.3	-	-	-	-
Gender						
Male	92	43.5	0.690	-	-	-
Female	43	44.2	-	-	-	-
Tumor size, mm						
≥50	57	33.3	0.007	-	-	-
<50	78	51.3	-	-	-	-
Tumor differentiation						
Well	31	54.8	0.013	-	-	-
Moderate	48	45.8	-	-	-	-
Poor	56	35.7	-	-	-	-
Pathological staging						
Early	23	87.0	<0.001	-	-	-
Advanced	112	34.8	-	-	-	-
Stage						
I	33	81.8	<0.001	1.700	1.287–2.244	<0.001
II	44	45.5	-	-	-	-
III	49	24.5	-	-	-	-
IV	9	0	-	-	-	-
Lymph node metastasis						
Negative	51	70.6	<0.001	0.596	0.372–0.956	0.032
Positive	84	27.4	-	-	-	-
RKIP methylation status						
Methylated	66	27.3	<0.001	1.602	1.038–2.473	0.033
Unmethylated	69	59.4	-	-	-	-
Protein expression of RKIP						
Negative	77	29.9	<0.001	-	-	-
Positive	58	62.1	-	-	-	-

RKIP, Raf kinase inhibitory protein; CI, confidence interval.
